# Prevalence of asthma among the adult general population of five Middle Eastern countries: results of the SNAPSHOT program

**DOI:** 10.1186/s12890-018-0621-9

**Published:** 2018-05-11

**Authors:** Hesham Tarraf, Omur Aydin, Dilsad Mungan, Mohammad Albader, Bassam Mahboub, Adam Doble, Aaicha Lahlou, Luqman Tariq, Fayaz Aziz, Abdelkader El Hasnaoui

**Affiliations:** 1 The Medical School, Cairo University, Cairo, Egypt; 2 Department of Chest Diseases, Division of Allergy and Immunology, Ankara University, School of Medicine, Ankara, Turkey; 3 Chest Medicine Department, Zain Hospital, Kuwait City, Kuwait; 4 College of Medicine, University of Sharjah, Sharjah, United Arab Emirates; 5 Department of Pulmonary Medicine, Rashid Hospital, Dubai, United Arab Emirates; 6 Foxymed, Paris, France; 7 MS Health, Rabat, Morocco; 8 GlaxoSmithKline, PO Box 50199, Dubai, United Arab Emirates

**Keywords:** Asthma, Prevalence, Middle East, SNAPSHOT, Quality of life, Co-morbidity

## Abstract

**Background:**

Asthma is a common chronic respiratory disease leading to morbidity, mortality and impaired quality of life worldwide. Information on asthma prevalence in the Middle East is fragmented and relatively out-dated. The SNAPSHOT program was conducted to obtain updated information.

**Methods:**

SNAPSHOT is a cross-sectional epidemiological program carried out in five Middle Eastern countries (Egypt, Turkey, Kuwait, Saudi Arabia, and the United Arab Emirates, the latter three grouped into a Gulf cluster) to collect data on asthma, allergic rhinitis, benign prostatic hyperplasia and bipolar disorder. The survey was carried out by telephone in a random sample of the adult general population with quotas defined according to country demographics. The analysis presented in this paper focuses on asthma. Subjects were screened for asthma based on criteria from the global Asthma Insights and Reality studies. Current prevalence (last 12 months) was estimated. Multivariate logistic regression analyses were used to investigate risk factors related to asthma and the association with allergic rhinitis and other co-morbidities. Quality of life was assessed using the three-level EQ-5D questionnaire.

**Results:**

2124 out of the 33,486 subjects enrolled in the SNAPSHOT program fulfilled the criteria for asthma. The adjusted prevalence of asthma ranged from 4.4% [95% CI: 4.0–4.8%] in Turkey, to 6.7% [95% CI: 6.2–7.2%] in Egypt and 7.6% [95% CI: 7.1–8.0%] in the Gulf cluster. Prevalence was higher (*p* < 0.0001) in women than men and increased with age (*p* < 0.0001). Co-morbidities occurred more frequently in asthma subjects compared to the non-asthma population (38% vs. 15% *p* < 0.0001). Subjects with asthma reported a lower (*p* < 0.0001) EQ-VAS score (68.2 ± 22.9) compared to the general population (78.1 ± 17.5). The risk factors associated with asthma were age, gender, country, and certain co-morbidities, namely respiratory, cardiovascular, gastrointestinal, nervous, and neurological diseases.

**Conclusion:**

The observed adjusted prevalence of asthma in the Middle East ranges from 4.4% to 7.6%, which is comparatively lower than the reported prevalence in Europe and North America. Asthma has a negative impact on quality of life, and is associated with high levels of co-morbid diseases, indicating a need for physicians to check for co-morbidities and ensure they are managed correctly in all asthma patients.

## Background

Asthma is one of the most common chronic respiratory diseases and is a major public health issue globally, affecting people of all ages, genders and ethnicities. It is estimated that the number of people with asthma worldwide may be as high as 334 million according to a report from the Global Asthma Network published in 2014 [1]. Prevalence has been shown to vary widely both between countries and within countries, and has been steadily increasing alongside that of allergy, as modern lifestyles are adopted and communities become more urbanised, a trend that is predicted to continue over the next two decades [2]. For those people affected by the disease, it can be a cause of major disability and impact greatly on quality of life [1, 2].

Asthma prevalence has been widely studied globally. However, many of the studies focus on the childhood prevalence of the disease, notably the International Study of Asthma and Allergies in Childhood (ISAAC) studies [3, 4]. In adults, three large, international, epidemiological projects assessing the prevalence of asthma have been conducted to date: the European Community Respiratory Health Survey (ECRHS) [5]; the World Health Survey (WHS) [6]; and the Global Allergy and Asthma Network of Excellence (GA2LEN) study in Europe [7]. Additional studies have estimated the prevalence and management of asthma in specific geographical areas such as the Asthma Insights and Reality (AIR) surveys [8], all of which utilise similar methodology and have now been carried out in a number of Middle Eastern countries including Turkey [9] and the Gulf [10]. However, to improve the understanding of asthma prevalence worldwide, further international studies are needed and require use of a consistent case definition for asthma and identical study methodologies. This would allow cross country comparison of data and provide a benchmark for future studies.

Despite the magnitude of the disease, data on the epidemiology and disease burden of asthma in the Middle East region are scarce. Local observational studies investigating the current prevalence of asthma in adults have been conducted, but few studies provide prevalence estimates at a multinational level using a standardised methodology and the data is relatively out-dated as the majority of the studies were completed prior to 2010. In Turkey, the published prevalence ranges from 2.11% - 8.35% [9, 11–15]. In the United Arab Emirates (UAE) the reported prevalence ranges from 2.79% - 8% [14, 16, 17] and in Saudi Arabia, a national household survey was carried out in 2013 which reported an asthma prevalence of 4.05% [18]. In the North African countries, the most recent data are from the AIR Maghreb (AIRMAG) study carried out between 2008 and 2009 [19], which investigated asthma prevalence in Algeria, Morocco and Tunisia. Earlier data (2002–2003) is available from the WHS survey [14]. In these countries, the reported prevalence rates ranged from 2.79% to 3.89%. In Egypt many studies on asthma have been performed in the paediatric age groups but very few addressed the adult population and were not representative of the whole country [20].

In some areas, there is limited regional information available. In addition, results from the existing studies rely on a variety of methodologies used to capture the information, the different population sources and variety of sampling methods, the different modes of interview, and the lack of a common case definition for asthma, and differing interpretation of symptoms in different countries all limit the ability to compare the data. Hence, there is a need to provide up to date information on the prevalence of asthma in Middle Eastern countries. To address this, we have conducted a large, cross-sectional, population-based study as part of the SNAPSHOT program, using a standardised methodology in five countries in the region, to investigate the current prevalence of asthma in the adult population.

## Methods

### The SNAPSHOT program

SNAPSHOT is a cross-sectional, observational, population-based program, comprising multiple studies, conducted in a random sample of the general population of five countries (Egypt, Kuwait, Saudi Arabia, Turkey, and the UAE). The objective of the SNAPSHOT program was to provide an omnibus approach to collect data simultaneously about multiple diseases using a standardised methodology. The program provides updated epidemiological data on the prevalence, burden of disease, quality of life and healthcare resource use related to four chronic diseases in the participating countries, namely: asthma, allergic rhinitis, bipolar disorder and benign prostatic hyperplasia (BPH). The selection of these diseases was based on the need to respond to emerging health technology assessment (HTA) requirements. The detailed study methodology is the subject of a separate manuscript [21].

The program was carried out by computer-assisted personal interviewing (CAPI) conducted over the telephone and the information collected via web-based electronic data capture. Interviews were proposed in Arabic, English or Turkish using translated validated questionnaires and conducted by trained staff of local specialised Contract Research Organisations (CROs); Omega (Turkey) for interviews in Turkey and Infomine Healthcare Research (Egypt) for interviews in the Arabic-speaking countries. Data management and analyses were performed by MS Health (Morocco).

### The SNAPSHOT sample

The SNAPSHOT population was generated using a random stratified sampling method. Enrolment began in July 2014. A target sample of 10,000 subjects from the adult general population of Turkey and Egypt and 15,000 for the Gulf cluster (comprised of Kuwait, Saudi Arabia and the UAE) was defined based on the demographic structure of the country in terms of age and gender, to enable the estimate of prevalence and disease burden with a satisfactory precision. In the Gulf cluster, the target sample size could not be reached despite extensive efforts to accelerate subject enrolment. Therefore, it was decided to stop recruitment in February 2016. The combined number of interviews conducted to date in Saudi Arabia, Kuwait and UAE showed that the program objectives could be achieved from a sample size perspective. The database was locked on 11th April 2016.

### Subject enrolment

Telephone numbers (landline and mobile) were generated in blocks using an assisted random-digit dialling procedure. Each number was dialled up to fifteen times on different days and times including weekends and evenings until contact was established. After fifteen attempts, outcomes were categorised as contact, unattributed, out-of-service, fax, or unreachable. In order to optimise response rates, successive blocks of numbers were defined and the release of a new block could only occur once the previous block had been completed. This process was repeated until the target number for each country / cluster was achieved.

**Fig. 1:**
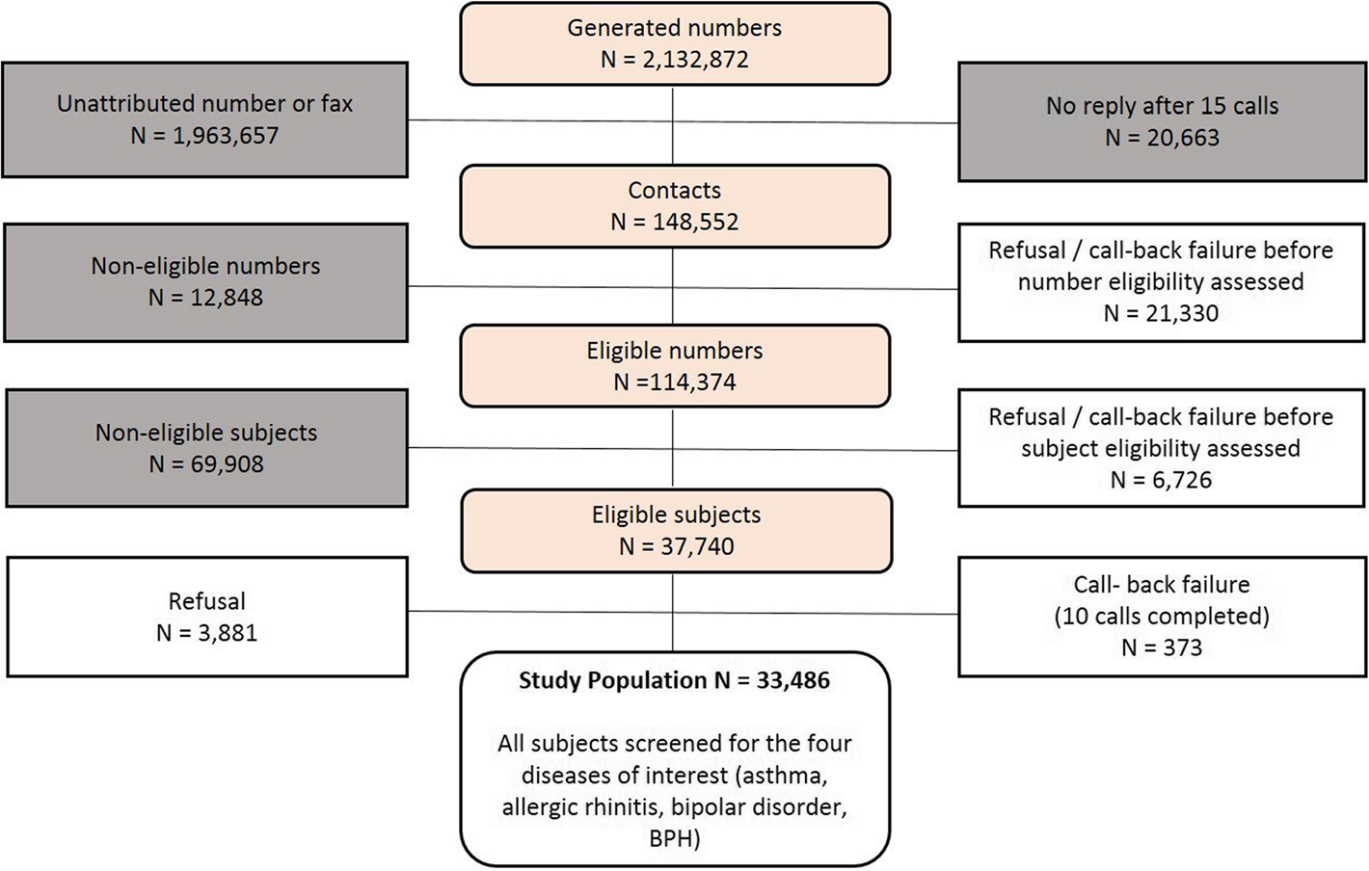
Subject flow through the recruitment process. The grey boxes indicate excluded numbers or subjects, and the white boxes the groups of potentially eligible numbers or subjects taken into account in the calculation of the response rate

If telephone contact was established, the interviewer first assessed the eligibility of the telephone number (business numbers were ineligible). For eligible numbers, the interviewer determined the respondent’s age, gender and region of residence. All subjects ≥18 years of age who had been residing in the country for over 6 months were eligible, unless the pre-specified strata for age, gender and region was filled. If the subject was ineligible, this was documented and the phone call terminated. If the subject was eligible, the interview began. If the respondent agreed to continue the interview but asked to be called back later, up to ten attempts were made after which this was classified as a call-back failure and considered a refusal. Subject flow through the recruitment process is shown in Fig. 1. The overall response rate was 50.9%. At a country level it varied by a factor of almost two, from 35.3% in Kuwait, 40.0% in UAE and 40.6% in Saudi Arabia to 58.9% and 67.4% in Turkey and Egypt respectively.

### Conduct of the interview

Standardised general information was provided at the start of the interview, specifying that participation was voluntary, confidential and anonymous. Verbal consent to participate was collected by the interviewee and recorded in the CAPI system and the interviewee was then invited to respond to a first questionnaire, screening for the four diseases of interest and to document social and demographic characteristics, as well as the presence of co-morbidities. If the interviewee did not want to answer, the contact was considered as a refusal and the interview terminated. If a subject screened positive for at least one of the four diseases, a second disease-specific questionnaire was administered to collect additional information on burden of disease, disease management and healthcare resource utilisation. In cases where respondents fulfilled the screening criteria for more than one disease, they were randomised by the CAPI system to respond to only one of the disease-specific questionnaires, in order to limit the duration of the interview. For all subjects the interview ended with the EuroQoL five-dimension questionnaire (EQ-5D), a measure of health status which captures the impact of a disease on physical, mental, and social functioning [22, 23]. All questionnaires were used in validated English, Turkish or Arabic translations; the translated versions are available on request. The full interview was conducted in one telephone call.

### Case definition for asthma

Subjects were considered to have asthma if they fulfilled the screening criteria shown in Table 1, which were based on the criteria used in the global AIR studies [8]. A positive answer to any of the latter three questions (Q3-Q5) led to classification of the interviewee as a subject with asthma. It is important to note that the criteria were designed to collect information on the current prevalence (last 12 months) and not the lifetime prevalence of the disease.

**Table 1:** Screening Criteria for Asthma. A positive answer to any of the latter three questions (Q3-Q5) led to classification of the interviewee as a subject with asthma

Screening Criteria for Asthma	
Q1 Have you been told by your doctor that you suffer from asthma?	□ No
	□ Yes
Q2 Have you had one of the following symptoms: wheezing, nocturnal coughing, chest tightness, or breathless ness in the last 12 months	□ No
	□ Yes
Q3. Have you had an asthma attack in the last 12 months?	□ No
	□ Yes
Q4. Have you used asthma medications in the last 12 months?	□ No
	□ Yes
Q5. Have you used Ventolin or inhaled bronchodilators or short acting β agonist in the last 12 months?	□ No
	□ Yes
If yes, what is the daily frequency of use?	□ Once
	□ Repeated

### Data collected for this analysis

This analysis focuses solely on asthma and aims to provide estimates of the current prevalence of asthma in the areas studied. Socio-demographic data were collected to describe the characteristics of the overall study population, including gender and age distribution, marital status, educational level, employment status, health system coverage, body mass index (BMI), the presence of co-morbidities and smoking status. If a respondent was a current or former smoker, they were asked to provide the duration and extent of exposure. Additionally, the type and frequency of co-morbidities were investigated and risk factors related to asthma identified. As part of the screening questionnaire, subjects were also screened for allergic rhinitis using the Score for Allergic Rhinitis (SFAR) questionnaire [24] and asked about any family history of this disease to identify asthma subjects who had co-morbid allergic rhinitis or a family history of allergic rhinitis. All subjects were also asked to complete the EQ-5D-3L questionnaire to measure quality of life, a generic questionnaire to measure health status. The questionnaire consists of five dimensions (mobility, self-care, usual activities, pain / discomfort, anxiety / depression) each of which can take one of three responses (no problems / moderate problems / extreme problems) and a visual analogue rating scale (EQ-VAS) [22]. Asthma prevalence data were collected using the case definition described and the prevalence by age, gender, country and region was assessed.

### Statistical analysis

The data presented here are for the screening population of 33,486 subjects who accepted to participate in the study, completed all screening questionnaires and thus constituted the screening population. Prevalence was adjusted for age and gender by weighting each subject to take into account the actual structure of age and gender in the national population for each country [25]. This adjustment also took into account the additional weight given to men over the age of 50 which was necessary to enable the prevalence of BPH to be estimated accurately.

Data are presented as proportions and means with standard deviations (SD), or medians with interquartile ranges (IQR). 95% confidence intervals (95% CI) were calculated for binomial data. Associations between categorical variables were estimated using the χ^2^ test and the Cochran–Mantel–Haenszel test as appropriate. Two-sided tests were used in all cases and a probability threshold of 0.05 was considered significant. Multivariate regression analysis was performed to identify risk factors associated with asthma. In a first step, variables were evaluated independently in a univariate analysis. All variables with a *P* value < 0.20 [26] in the univariate analysis were entered into the multiple logistic regression analysis in which variables were retained in the model using a backward stepwise selection to determine the variables associated with an increased risk of asthma at the probability level of 0.05. In addition, variables hypothesised to have clinical relevance but not found to be significant at 0.20 were maintained (e.g. age). A final multivariate analysis was conducted to generate odds ratios. Multivariate regression analysis was also performed to assess the relationship between co-morbidities and asthma independent of age, gender and country and the relationship between allergic rhinitis and asthma. All statistical analyses were performed using SAS® Version 9.4 (SAS, Cary, USA).

## Results

### Study sample

A total of 33,486 subjects agreed to participate in the screening questionnaire and constituted the screening population. This population was distributed between Egypt (10,014), Turkey (10,000) and the Gulf cluster (13,472). Overall, approximately 60% of respondents were men and 40% women (sex ratio: 1.41; 19,610 were men). The majority were aged between 18 and 35 (47.7%; *n* = 15,959). Just over half of the remaining respondents were between 35 and 50 years of age (29.6%; *n* = 9921) and the rest were aged 50 or above (22.7%; *n* = 7606). Selected demographics of the screening population are shown in Table 2. These data highlight that the majority of the screening population are non-smokers (65%) and either overweight or obese (54%).

**Table 2:** Demographics of the screening population

Demographic	Criteria	Egypt	Gulf cluster	Turkey
		*N* = 10,014	*N* = 13,472	*N* = 10,000
Gender *n* (%)	Count	10,014	13,472	10,000
	Men	5747 (57.4)	8524 (63.3)	5339 (53.4)
	Women	4267 (42.6)	4948 (36.7)	4661 (46.6)
Age *n* (%)	Count	10,014	13,472	10,000
	18–34 years	4978 (49.7)	7055 (52.4)	3926 (39.3)
	35–49 years	2453 (24.5)	4745 (35.2)	2723 (27.2)
	≥ 50 years	2583 (25.8)	1672 (12.4)	3351 (33.5)
Marital Status *n* (%)	Count	9814	13,013	9522
	Married	7357 (75.0)	9000 (69.2)	7054 (74.1)
	Living as unmarried couple	3 (0.03)	4 (0.03)	6 (0.06)
	Separated/divorced	160 (1.6)	325 (2.5)	130 (1.4)
	Widowed	362 (3.7)	206 (0.02)	390 (4.1)
	Single/never married	1932 (19.7)	3478 (26.7)	1942 (20.4)
Education level *n* (%)	Count	9755	12,877	9079
	No high school	2767 (28.4)	1789 (13.9)	5245 (57.8)
	Some high school	236 (2.4)	283 (1.2)	156 (1.7)
	High school graduate	2291 (23.5)	3501 (27.2)	1842 (20.3)
	Technical post-secondary	1386 (14.2)	834 (6.5)	306 (3.4)
	Some college	434 (4.5)	757 (5.9)	430 (4.8)
	College graduate	2267 (23.2)	4837 (37.5)	1040 (11.5)
	Post graduate degree	374 (3.8)	876 (6.8)	60 (0.7)
Employment Status *n* (%)	Count	9735	12,834	9721
	Employed (full or part time)	5592 (57.4)	8732 (68.0)	4299 (44.2)
	Unemployed	447 (4.6)	584 (4.6)	1243 (12.8)
	Retired and not working	579 (6.0)	291 (2.3)	1462 (15.0)
	Student	524 (5.4)	738 (0.06)	340 (3.5)
	Homemaker	2545 (26.1)	2466 (19.2)	2370 (24.4)
	Disabled or too ill to work	48 (0.5)	23 (0.2)	7 (0.07)
Yearly Household Income *n* (%)	Count	9.596	12,555	9125
	< minimum wage	3465 (36.1)	1616 (12.9)	1427 (15.6)
	Minimum wage	4110 (42.8)	8917 (71.0)	3023 (33.1)
	2 × minimum wage	1277 (13.3)	1243 (9.9)	4276 (46.9)
	> 2 times minimum wage	744 (7.8)	779 (6.2)	399 (4.4)
Smoking *n* (%)	Count	9814	13,014	9784
	Non-smoker	6513 (66.4)	9176 (70.5)	5942 (60.7)
	Current/former smoker	3301 (33.6)	3838 (29.5)	3842 (39.3)
BMI *n* (%)	Count	9338	12,156	9028
	Underweight	230 (2.5)	469 (3.9)	271 (3.0)
	Normal weight	3060 (32.8)	4605 (37.9)	3826 (42.4)
	Overweight	3253 (34.8)	4333 (35.6)	3299 (36.5)
	Obese	2795 (29.9)	2749 (22.6)	1632 (18.1)

Demographics of the screening population (*N* = 33,486) by country and cluster. BMI = body mass index

### Current prevalence of asthma

Of the subjects enrolled in the study 2124 fulfilled the case definition for asthma and were defined as the asthma population. Overall, the adjusted prevalence of asthma in the adult general population over 18 years of age in the countries studied was 6.4% [95% CI: 6.1–6.6%]. This ranged from 4.4% in Turkey, to 6.7% in Egypt and 7.6% in the Gulf cluster (Table 3). After adjusting for age and gender there is little difference between the crude and adjusted prevalence. The adjusted prevalence by country in the Gulf cluster was 9.5% [95% CI: 8.1–10.9%] in Kuwait, 4.9% [95% CI 4.2–6.6%] in the UAE, and 8.3 [95% CI 7.7–8.9%] in Saudi Arabia.

**Table 3:** Current prevalence of asthma

Variable		Egypt	Gulf cluster	Turkey
		N = 10,014	N = 13,472	N = 10,000
Number of Cases		676	1003	445
Crude Prevalence (%)		6.8	7.5	4.5
Adjusted Prevalence (%)		6.7	7.6	4.4
Adjusted 95% CI		[6.2–7.2]	[7.1–8]	[4.0–4.8]
Gender (%) [95% CI]	Men	5.5 [4.9–6.1]	6.2 [5.7–6.7]	2.9 [2.4–3.3]
	Women	8.4 [7.6–9.3]	9.6 [8.7–10.4]	6.3 [5.6–7]
	*P* value (CMH test)	*p* < 0.0001		
Age (%) [95% CI]	18–34	5.5 [4.8–6.1]	7.0 [6.4–7.6]	2.6 [2.1–3.1]
	35–49	7.7 [6.7–8.8]	7.7 [7–8.5]	4.0 [3.3–4.8]
	≥ 50	8.3 [7.3–9.4]	8.7 [7.3–10.0]	7.0 [6.1–7.8]
	*P* value (CMH test)	*p* < 0.0001		

Current prevalence (%) of asthma by country and cluster, adjusted by age and gender as required. CMH: Cochran Mantel-Haenszel; 95% CI = 95% Confidence Interval

The current prevalence of asthma was significantly higher in women compared to men across all countries and the cluster of countries studied (*p* < 0.0001). Age is also an important factor; the current prevalence of asthma increased significantly with age (*p* < 0.0001). These results are presented in Table 3. Further analysis of the age and gender split by country showed that for each country or cluster of countries the observed prevalence of asthma was higher in women than in men across every age group studied (data not shown).

**Fig. 2:**
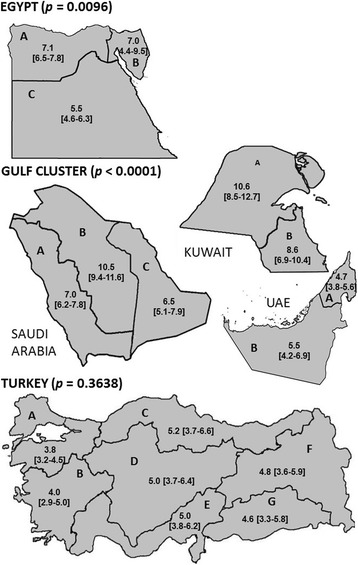
Prevalence of asthma by region. Prevalence of asthma (% [95% CI]) by region across the countries studied *P value (χ^2^ test); adjusted by age and gender. Egypt: A = Greater Cairo/North Egypt, B = Canal/other, C = Upper Egypt. Kuwait: A = Western Kuwait, B = Eastern Kuwait. UAE: A = East UAE, B = Abu Dhabi. Saudi Arabia: A = Western Saudi Arabia, B = Central Saudi Arabia, C = Eastern Saudi Arabia. Turkey: A = Marmara, B = Aegean, C = Black Sea, D = Central Anatolia, E = Mediterranean, F = Eastern Anatolia, G = South Eastern Anatolia. Note: These maps were prepared using the country outline maps available in Wikimedia Commons under the GNU Free Documentation License and adapted by the authors to include the prevalence data

Current asthma prevalence was also investigated by region across the countries studied and these results are shown in Fig. 2. In Egypt, the highest prevalence was documented in Greater Cairo / North Egypt, and the Canal/other region followed by Upper Egypt (*p* < 0.0096). In the Gulf cluster, there was a significant difference in prevalence across the countries and regions studied (*p* < 0.0001). In Saudi Arabia, the central region reported a prevalence of 10.5% which is significantly higher than the coastal regions in the east and west which reported a prevalence of 6.5% and 7.0% respectively. The prevalence in Kuwait was comparatively high compared to most other regions in the Gulf cluster although the reported prevalence in western Kuwait (10.6%) was in line with that seen in central Saudi Arabia. The prevalence in the UAE was slightly lower than the rest of the Gulf cluster but consistent across the country. In Turkey, there was no significant difference in prevalence across the regions studied (*p* = 0.36).

### Asthma and smoking status

There was no statistical difference (*p* = 0.99) in the percentage of respondents who smoked between the asthma population (66.5%) and the non-asthma population (66.3%). Among current or former smokers, there was no statistical difference (*p* = 0.08) in the number of pack-years for the asthma population (18.9 ± 23.0) compared to the non-asthma population (17.3 ± 20.4).

### Risk factors for asthma

**Fig. 3:**
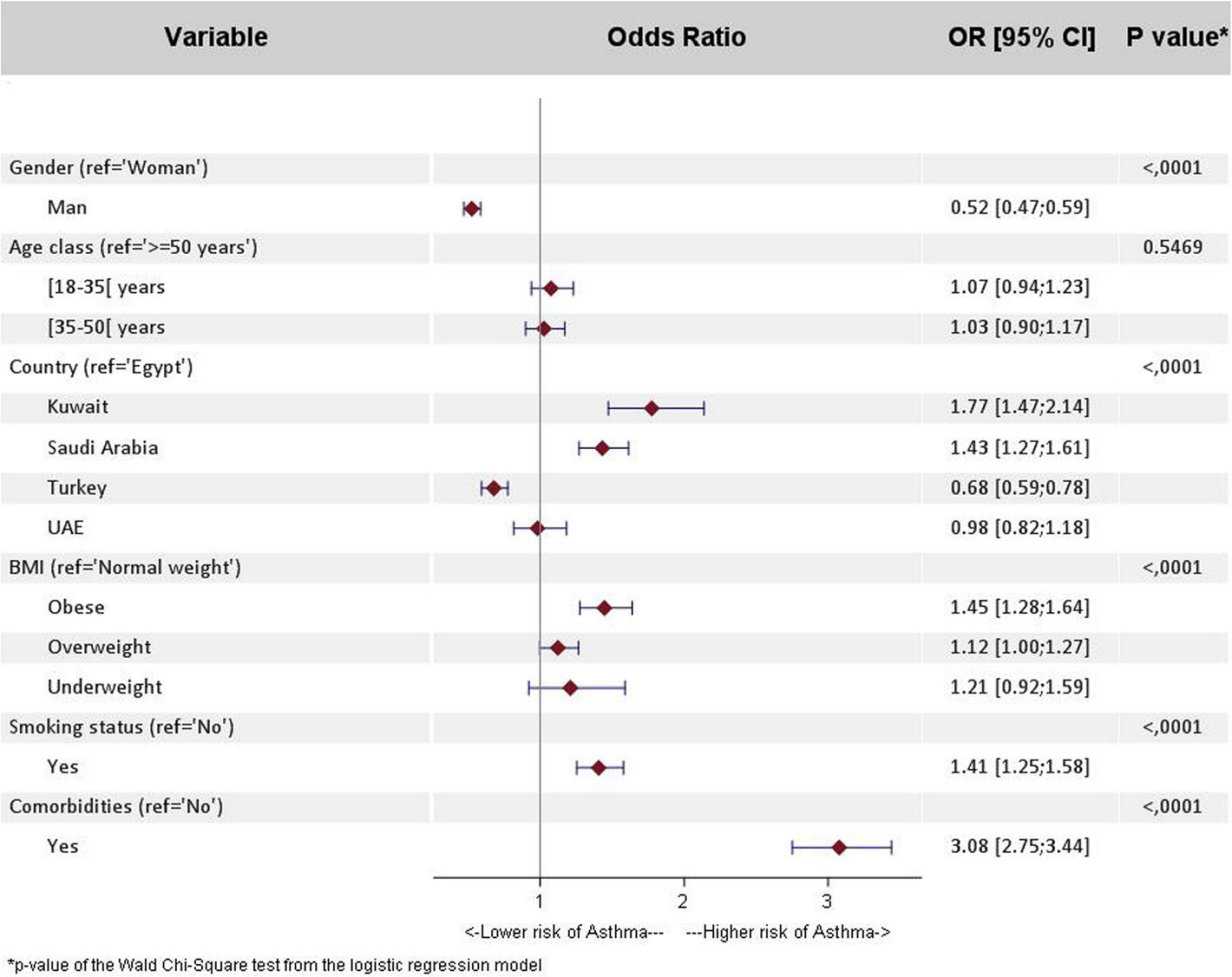
Multivariate regression analysis investigating the risk factors for asthma: Asthma population (1931 subjects) versus non-asthma population (28,586 subjects); OR [95% CI] = odds ratio [95% Confidence Interval]. The results presented are adjusted for age (18–34,35–49, ≥50 years), gender and country (Egypt, Kuwait, Saudi Arabia, Turkey, UAE)

The following variables were tested in a univariate analysis looking at potential risk factors for asthma: marital status, employment status, BMI, smoking status, co-morbidities yes/no, age, gender and country (Egypt, Kuwait, Saudi Arabia, Turkey, UAE). Those variables found to be significant at 0.2 were entered into the multivariate analysis. The variable smoking status was not found to be significant. However, to further explore a potential association between smoking and asthma this variable was maintained in the multivariate analysis. The results of a multivariate regression analysis show a significant association between living in Kuwait or Saudi Arabia and having asthma (odds ratio (OR) 1.8 and 1.4 respectively) compared to living in Egypt (*p* < 0.0001). In addition, a significant association (*p* < 0.0001) was observed between asthma and obesity (OR 1.5), smoking (OR 1.4), and the presence of co-morbidities (OR 3.1). These results are shown in Fig. 3.

### History of co-morbidities and the impact on the asthma population

**Fig. 4:**
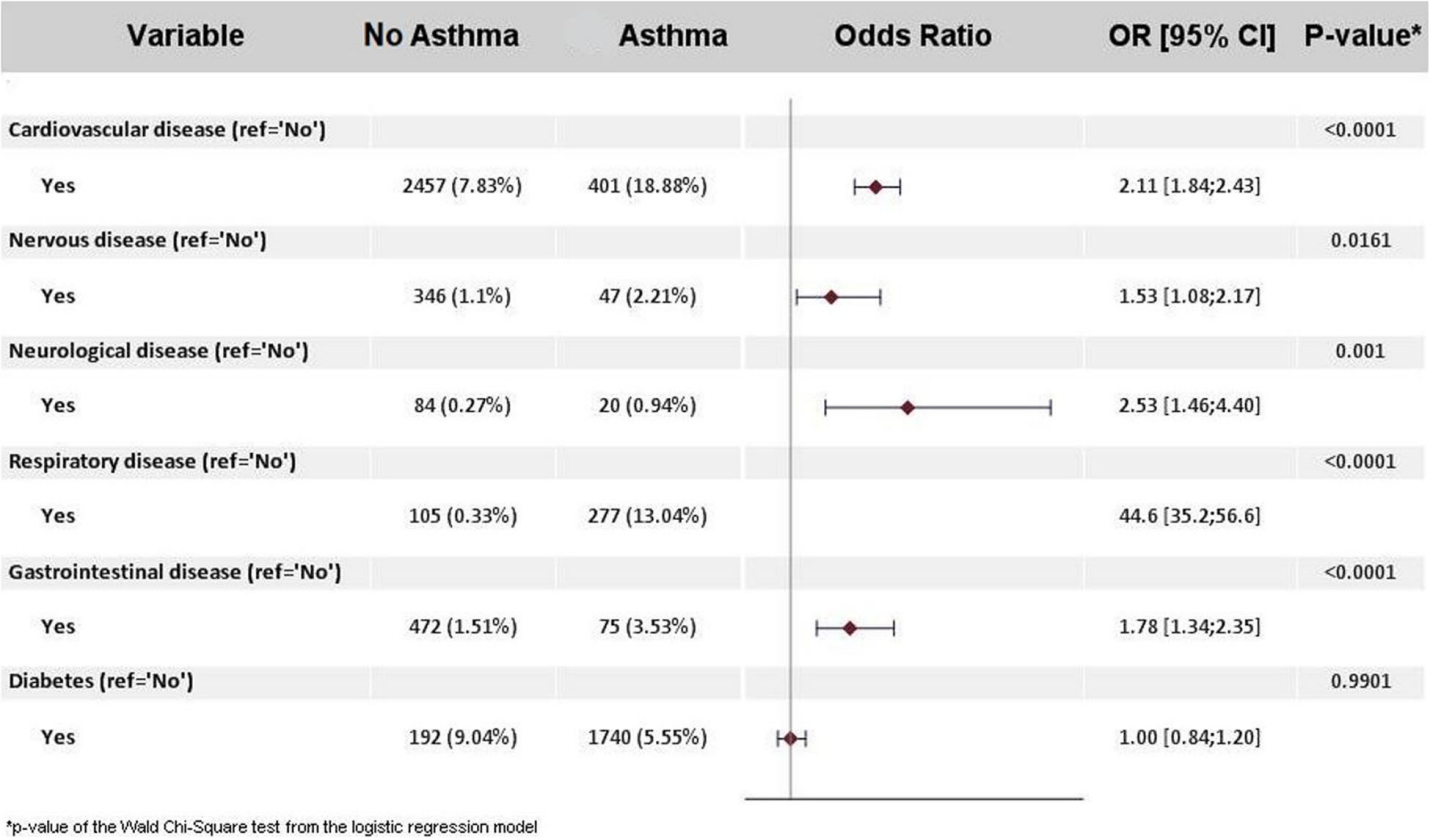
Multivariate regression analysis investigating the impact of co-morbidities on the risk of asthma. Asthma population (2124 subjects) versus non-asthma population (31,362 subjects); OR [95% CI] = Odds Ratio [95% Confidence Interval]. The results presented are adjusted for age (18–34,35–49, ≥50 years), gender and country (Egypt, Kuwait, Saudi Arabia, Turkey, UAE)

The number of subjects that reported suffering from a chronic health condition was significantly higher (*p* < 0.0001) in the asthma population (38.4%) compared to the non-asthma population (15.4%). The following variables were tested in a univariate analysis looking at the relationship between co-morbidities and asthma: cardiovascular disease, nervous disease, neurological disease, respiratory disease, gastrointestinal disease, renal disease, rheumatological disease, endocrine disease (diabetes), malignancy disease, immunological disease and adjusted for age, gender and country (Egypt, Kuwait, Saudi Arabia, Turkey, UAE). Those found to be significant at 0.20 were entered into a multivariate analysis to investigate the relationship between each of the co-morbidities and the risk of asthma, independent of age, gender and country. The results show that the co-morbidity with the highest impact is respiratory disease (OR 44.6). Detailed investigation into the conditions classified under the co-morbidity ‘respiratory disease’ revealed that this refers primarily to chronic obstructive pulmonary disease (COPD). This was followed by neurological disease (OR 2.5), cardiovascular disease (OR 2.1), gastrointestinal disease (OR 1.8) and nervous disease (OR 1.5). These results are presented in Fig. 4.

A large number of subjects with asthma (482) were found to have co-morbid allergic rhinitis and 271 subjects reported having a family history of the disease, although they did not have allergic rhinitis themselves. A multivariate regression analysis looking specifically at the relationship between allergic rhinitis and asthma, adjusted for age, gender and country showed a strong association (*p* < 0.0001) between the two diseases (OR 6.4; 95% CI 5.7–7.2). In case of a family history of allergic rhinitis in subjects without allergic rhinitis themselves a weaker association with asthma was observed (OR 1.7; 95% CI 1.0–3.0).

### Impact of asthma on quality of life

**Fig. 5:**
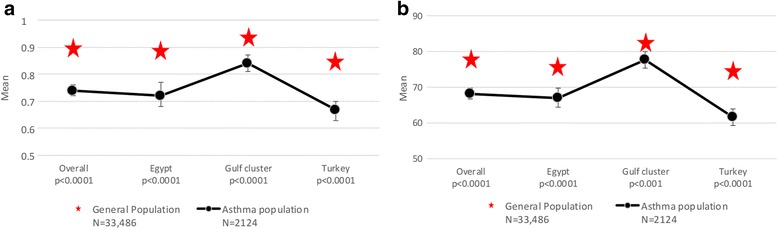
Impact of asthma on quality of life. Quality of life was assessed using the three level EuroQol five-dimension questionnaire (EQ-5D-3L). Comparison of the EQ-5D-3L utility values (**a**) and EQ-VAS scores (**b**) between the asthma population and general population by country or cluster are shown. **a** For the asthma population (*n* = 2124) the data represent the mean EQ-5D-3L utility value with the 95% CI. For the general population (*N* = 33,486), the mean EQ-5D-3L utility value is presented. **b** For the asthma population (n = 2124) the data represent the mean EQ-VAS score with the 95% CI. For the general population (*n* = 33,486), the mean EQ-VAS score is presented

Overall, subjects with asthma reported a significantly lower (p < 0.0001) mean EQ-5D-3L utility score (0.74 ± 0.34) than the general population (0.90 ± 0.21). This relationship was observed for all participating countries or cluster of countries. A similar observation was made for the mean EQ-VAS scores (68.2 ± 22.9 in subjects with asthma; 78.1 ± 17.5 in the general population p < 0.0001). The overall impact and the country-level results are presented in Fig. 5.

## Discussion

Omnibus programs such as SNAPSHOT represent a powerful approach for generating standardised epidemiological data on important health indicators such as prevalence. In particular, the use of a common methodology to collect the data enables pertinent comparisons to be made between countries and between diseases. Although setting up multidimensional studies such as this on a large scale is time- and resource- consuming, once the data acquisition system has been validated it would be relatively straightforward to implement the same program in further groups of countries.

In this program the overall response rate was 50.9%. The SNAPSHOT program was conducted in a random selection of the general population using telephone interview. In this context, a high level of refusal is common since people often do no do not respond to such calls requesting information, from which they receive no immediate benefit. However, the combination of refusals and call-back failures, and the inclusion of subjects prior to confirmation of eligibility criteria in the response rate calculation, reflects a conservative approach. If we assume that the distribution of eligible/non-eligible subjects is identical for respondents and non-respondents, then the response rate could be estimated as 56.5%. Further exclusion of call-back failures from the calculation would have yielded a response rate of 70.6%. If this non-conservative response rate is used then the response rate for the SNAPSHOT program is within the range of that observed in AIR studies elsewhere, such as Europe (80%) [27], and the Maghreb [19].

The primary objective of this study was to estimate the prevalence of asthma in five countries in the Middle East (Egypt, Turkey Kuwait, Saudi Arabia, and the UAE). The overall current prevalence of asthma in the adult general population over 18 years of age in these countries was 6.4%. This ranged from 4.4% in Turkey, to 6.7% in Egypt and 7.6% in the Gulf Cluster.

The global AIR studies have been conducted in many countries, and although these studies use the same questionnaire, a very broad range of prevalence estimates has been observed in the different studies conducted (ranging from 1% in Ecuador to 15% in Singapore) [28]. Comparison of the reported prevalence of asthma in these Middle Eastern countries with data obtained through the AIR studies demonstrates that the prevalence of asthma in the participating countries is low compared with the United Kingdom but slightly higher than some other European countries such as Germany [27], and considerably higher than countries in Asia such as China and Malaysia [29].

Comparison with published epidemiological data on the prevalence of asthma in the Middle East is difficult, since the criteria used to define asthma vary between studies and the methodologies are not consistent. For example, the studies use different case definitions for asthma; different populations, different modes of interview; or different age cut-offs for inclusion. This has led to a range of prevalence estimates being reported. One of the objectives of the SNAPSHOT program was to use a consistent case definition and study methodology across all countries included, to overcome this issue and enable comparison of the results.

**Fig. 6:**
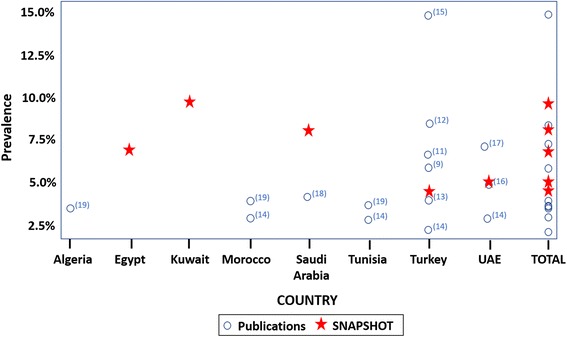
Asthma prevalence in the Middle East. Prevalence of asthma as reported by SNAPSHOT and earlier studies in the Middle East and North Africa region. The inclusion criteria for the literature review were: article published between January 2000 and December 2015, population-based observational studies investigating the current prevalence of asthma in adults. The red star denotes the prevalence findings from the SNAPSHOT study. Note: two Turkish studies [9, 11] were carried out in urban centres only and were not national studies

To gain a better understanding of previously published asthma prevalence data in the region, a literature review was performed. This review focused on general population studies which investigated asthma prevalence in the Middle East and North Africa region. In line with the parameters of the SNAPSHOT study, the search was limited to population-based, observational studies that focused on the current prevalence of asthma in adults. The search was restricted to articles that were in English, available via PubMed, and were published between January 1st 2000 and December 31st 2015 [9, 11–19]. The findings, together with the results from the SNAPSHOT study (denoted by a red star), are depicted in Fig. 6. The reported prevalence of asthma in Turkey and the UAE is within the range of the published literature and there are no published studies that fulfil the specified inclusion criteria in Egypt and Kuwait. A higher prevalence of asthma was observed in Saudi Arabia compared to the previously published data. This could be due to methodological differences between the studies, (the interviews in the previous study had been conducted face-to-face which tends to yield lower prevalence estimates than telephone or postal questionnaires), although a real increase in prevalence over time cannot be excluded, perhaps linked to the increasing urbanisation of the country [30].

Whilst the prevalence of asthma in Turkey reported here is within the range reported by comparable studies in the country, the prevalence reported in Turkey was generally lower than in Egypt or the Gulf cluster, the reported prevalence in the 18–34 age group was particularly low at 2.6%. There are few published studies that have investigated asthma prevalence by age in Turkey, and none using equivalent methodology to SNAPSHOT. However, a study carried out in the city of Samsun reported the prevalence by asthma diagnosis as 1.0% in the 15–29 age group and 2.6% in the 30–49 age group, and by use of medication as 1.0% and 1.8% in these respective age groups [31]. This is in line with the findings presented here.

It has been documented that the prevalence of allergic disease is generally higher in urban compared to rural areas [32]. Consistent this with theory, the United Nations (UN) 2014 World Urbanisation report indicates that the Gulf countries that participated in this study have a higher urban population percentage than Turkey and Egypt, and this cluster reports the highest prevalence of asthma. However, The UN report estimates 43% of the Egyptian population are urban compared to 73% of the Turkish population, yet the reported prevalence of asthma in this study is higher in Egypt compared to Turkey [33]. Data from our study also describes regional variations in prevalence that cannot be explained solely by urbanisation. For example, whilst the regional asthma prevalence reported in Kuwait could correlate with the presence of increased urban extents as defined in the Global Rural-Urban Mapping Project (GRUMP) [34], this is not the case for Saudi Arabia, where the highest urban extents are reported in the western region [35], yet the highest asthma prevalence in this study was reported in central Saudi Arabia. The variations in prevalence between countries and regions reported in the SNAPSHOT study could be influenced by population density and urbanisation. However, these may not be the only factors involved; for example, elements such as air pollution, climate exposure and allergens could play a role.

Many studies have consistently shown a higher prevalence of asthma in women during adulthood [36, 37]. Consistent with this, the current prevalence of asthma reported in SNAPSHOT was higher among women compared to men, a trend observed across all the countries studied. Multiple hypotheses have been put forward to explain this difference. For example, it has been reported that the prevalence of asthma increases significantly after puberty for women and female sex hormones may play a role [38]. However, to date no single explanation can fully explain the differences observed.

Smoking is considered a major risk factor for several diseases, one of which is COPD [39], and COPD often presents with similar symptoms to asthma. This can make a differential diagnosis of asthma more challenging, particularly in general population surveys like SNAPSHOT with lay interviewers and no case ascertainment by a physician. There was no significant difference between the numbers of smokers in the asthma population compared to the non-asthma population, suggesting little or no contamination of this population with COPD patients who have been wrongly classified as subjects with asthma, and supporting the case definition of asthma used in the SNAPSHOT study. Interestingly, despite the fact that there was no difference in the prevalence of asthma between smokers and non-smokers in this study, smoking was identified as a risk factor for asthma. One factor contributing to this could be that that the prevalence of smoking in the Middle East and North Africa is higher in men, as reported in the BREATHE study [40], whereas the prevalence of asthma is higher in women as reported here.

This study shows an association between certain co-morbidities and asthma, particularly allergic rhinitis (OR 6.4). The association between allergic rhinitis and asthma is supported in the literature [41]. The World Health organisation (WHO), through the Allergic Rhinitis and its Impact on Asthma (ARIA) program, has examined the impact of allergic rhinitis on asthma and concluded that both allergic and non-allergic rhinitis should be considered risk factors for asthma [42].

Overall, 54% of the screening population and 67.5% of the asthma population are overweight or obese. This is high compared to the global estimate from the WHO in 2014 which stated that 39% of adults aged 18 years and over were overweight and 13% of those were obese [43]. However, data from the 2013 Global Burden of Disease study demonstrates a higher prevalence of obesity in the MENA region compared to the global average [44]. An association between obesity and asthma has previously been reported; a meta-analysis of prospective epidemiological studies reported that those overweight were 38% more likely to develop asthma, rising to 92% of those who were obese [45]. Given the high incidence of obesity in the screening population the impact of cardiovascular disease is not unexpected since obesity predisposes subjects to cardiovascular disease. However, cardiovascular disease has also been linked to asthma independently. For example, a cohort study carried out in Northern California reported an association between asthma and increased risk of cardiovascular disease in women [46]; a large cross-sectional population-based study carried out in Adelaide, Australia also showed an association between asthma and cardiovascular disease independent of BMI, in a representative population sample aged 18 years and above [47]. An association between gastrointestinal disease and asthma was also reported in the SNAPSHOT study. Again, this could be linked to the high levels of obesity in the screening population and associated predisposition to gastro-oesophageal reflux disease (GERD). Indeed, there are numerous studies demonstrating an association between asthma and GERD. One such study has shown an independent association of GERD with poorly controlled asthma [48].

In addition to the co-morbidities mentioned above a significant correlation was also observed for nervous and neurological disease. Previously published studies have documented an association between asthma and diseases of the nervous system such as cerebrovascular disease. For example, the Atherosclerosis Risk in Communities (ARIC) study reported an association between asthma and stroke. Interestingly however, this study did not find an association between asthma and cardiovascular disease [49]. Neurological diseases such as dementia have also been previously linked to asthma. For example, a retrospective cohort study carried out in Taiwan reported an increased risk of developing dementia for those people with asthma compared to without asthma within a follow up period of 11 years [50]. The high levels of co-morbidities identified in the asthma population indicate that physicians need to check for co-morbidities as part of routine care and ensure they are managed correctly in all asthma patients as these conditions may influence asthma control.

Asthma is a condition that is known to have a negative impact on quality of life for those that suffer from the disease. Several disease specific questionnaires have been developed to assess the quality of life of patients with respiratory disease [51] and specifically asthma [52–56]. Both specific and generic quality of life measures [22, 57] have been used in a clinical setting to monitor the quality of life of asthma patients, and have demonstrated that asthma impairs quality of life with a more pronounced impact during exacerbations and in uncontrolled disease [58, 59]. However, general population studies investigating the effects of asthma on quality of life are scarce. The EQ-5D questionnaire is a widely used generic measure of health-related quality of life and is not specific to a particular disease, thus enabling comparisons between different disease areas. In addition, it is a brief, simple measure for patients to understand over the telephone in a general population setting. Previous studies with this questionnaire have demonstrated an impairment of quality of life in patients with respiratory disease [60]. The results of the SNAPSHOT study indicate that, across all countries studied, subjects with asthma suffer significant impairment in their quality of life. Whilst this observation is expected, it is the first time it has been shown in these countries on such a large scale in a general population setting.

The study has several strengths. It is a cross sectional, population-based study with a large sample size using consistent methodology across all countries, providing a standardised measure of prevalence in the Middle East. In addition, the case definition of asthma used is based on the global AIR surveys, and quality of life has been assessed using the EQ-5D questionnaire, which have been validated and used widely, enabling comparison with data collected elsewhere.

As with all studies, limitations do exist. These include the fact that the survey was telephone based, which could introduce a sampling bias if certain groups do not have access to a telephone, such as those in more remote, rural areas. However, the emergence of mobile telephones means that the majority of households in these countries have access to a telephone, and the SNAPSHOT program included both mobile and fixed landline telephone numbers. Another limitation was that the survey was conducted by trained lay interviewers; therefore, the diagnosis of asthma or other reported co-morbidities was not confirmed by a physician. In addition, reporting of a physician diagnosis of asthma was not one of the inclusion criteria for a positive screen. It is well-known that access to healthcare infrastructure and primary care is lower in the Middle East compared to Europe. Therefore, if the definition of asthma had been restricted to those subjects who had a confirmed asthma diagnosis by a physician, it would have excluded many subjects with undiagnosed asthma, as these subjects might experience issues around access to healthcare. The proportion of people with undiagnosed asthma is thought to be higher in this region compared to Europe. However, as part of the screening questionnaire, subjects were asked whether they had been told by a physician that they had asthma, and of the 2124 subjects with asthma included in the SNAPSHOT program, 70.2% (*n* = 1490) had a confirmed asthma diagnosis, validating the study findings based on the screening criteria used. As is the case for all studies that require participants to recall data, there is a risk of inaccuracy in the data collected. However, this is minimised by setting the inclusion criteria for the asthma population based on exacerbations and treatments over the last 12 months as opposed to simple symptom reporting.

## Conclusion

This study provides an updated prevalence estimate for asthma within the adult general population of five countries in the Middle East region, using an identical methodology across all countries studied. The reported prevalence estimates suggest that the Middle East is a low/medium prevalence area compared to other regions in the world. Prevalence is higher among adult women than men and increases with age. A positive correlation was observed for co-morbidities such as allergic rhinitis. In addition, asthma was shown to have a negative impact on quality of life; the first time this has been shown in these countries through a large scale general population based study. The lack of reliable recent data on the prevalence and burden of disease is a major obstacle to the implementation of effective public health strategies for disease prevention and management. The results from this study can contribute to informed decision making when setting priorities public health policy and strategy to manage asthma in these countries.

## Abbreviations

AIR: Asthma insights and reality
AIRMAG: Asthma insights and reality Maghreb
ARIA: Allergic rhinitis and its impact on asthma
ARIC: Atherosclerosis risk in communities
BMI: Body mass index
BPH: Benign prostatic hyperplasia
CAPI: Computer-assisted personal interviewing
CI: Confidence interval
COPD: Chronic obstructive pulmonary disease
CRO: Contract research organisation
ECRHS: European community respiratory health survey
EQ-5D: EuroQoL five dimension questionnaire
EQ-5D-3 L: 3 level EQ-5D questionnaire
EQ-VAS: EuroQol visual analogue scale
GA2LEN: Global allergy and asthma network of excellence
GEP: Good epidemiological practice
GERD: Gastro-oesophageal reflux disease
GRUMP: Global rural-urban mapping project
HTA: Health technology assessment
IQR: Inter quartile range
ISAAC: International study of asthma and allergies in childhood
OR: Odds ratio
SD: Standard deviation
SFAR: Score for allergic rhinitis
SFDA: Saudi food and drug authority
UAE: United Arab Emirates
UN: United Nations
WHO: World health organisation
WHS: World health survey
